# Supplementary material for: Associations Between Neonatal Cry Acoustics and Visual Attention During the First Year
Source: Front Psychol. 2020 Sep 30;11:577510. doi: 10.3389/fpsyg.2020.577510 (PMC7561366; doi:10.3389/fpsyg.2020.577510)
Supplement: Supplementary file 1 [file Data_Sheet_1.pdf]

Codebook

**Variable**	**Description**
code	Participant code (CT = Cape Town, Tre = Tampere)
SSRI	1 = mothers with SSRI medication in the Cape Town cohort, 0 = mothers without SSRI medication in the Cape Town cohort
CryFromBeg	Variable denoting whether the cry sample was captured from the beginning of the cry bout in the Tampere cohort, 1 = from the beginning, 0 = not from the beginning
F0	Mean of the F0means of the first 5 expiratory cry phases
F0var	Mean of the F0 standard deviations of the first 5 expiratory cry phases
Disengagement	Mean of dwell time indexes for the control stimulus, happy face, and fearful face
OculomotorOrienting	Oculomotor orienting latency
